# Detection of fickle trolls in large-scale online social networks

**DOI:** 10.1186/s40537-022-00572-9

**Published:** 2022-02-19

**Authors:** Hossein Shafiei, Aresh Dadlani

**Affiliations:** 1 Faculty of Computer Engineering, K. N. Toosi University, Tehran, Iran; 2 School of Engineering and Digital Sciences, Nazarbayev University, Nur-Sultan, Kazakhstan

**Keywords:** Online social networks, Large-scale networks, Troll detection

## Abstract

Online social networks have attracted billions of active users over the past decade. These systems play an integral role in the everyday life of many people around the world. As such, these platforms are also attractive for misinformation, hoaxes, and fake news campaigns which usually utilize social trolls and/or social bots for propagation. Detection of so-called social trolls in these platforms is challenging due to their large scale and dynamic nature where users’ data are generated and collected at the scale of multi-billion records per hour. In this paper, we focus on fickle trolls, i.e., a special type of trolling activity in which the trolls change their identity frequently to maximize their social relations. This kind of trolling activity may become irritating for the users and also may pose a serious threat to their privacy. To the best of our knowledge, this is the first work that introduces mechanisms to detect these trolls. In particular, we discuss and analyze troll detection mechanisms on different scales. We prove that the order of centralized single-machine detection algorithm is $O(n^3)$ which is slow and impractical for early troll detection in large-scale social platforms comprising of billions of users. We also prove that the streaming approach where data is gradually fed to the system is not practical in many real-world scenarios. In light of such shortcomings, we then propose a massively parallel detection approach. Rigorous evaluations confirm that our proposed method is at least six times faster compared to conventional parallel approaches.

## Introduction

Online social platforms have become an essential part of human interactions over the past few years. These platforms have attracted billions of people from around the globe. Recent studies showed that over 75% of users check their social pages at least once a day, and an average user spent 2 hours and 24 minutes per day on social media in 2020 [1]. It has also been shown that more than half of US citizens get their news from social media [2]. The dominant role of these platforms also attracts misinformation, hoaxes, and fake news campaigns that can propagate readily as factual information. For example, [3] showed that during the Ebola crisis fake news spread as quickly as accurate information on the Twitter social media platform. This rate of spread was (in part) facilitated by social trolls.

Social trolling is a general term coined to describe various types of disruptive behavior in social platforms, such as impersonating as experts (in the topic of discussion) and then propagating misinformation or fake news [4]. The act of trolling can be carried out by either a real person or a social bot which essentially, is a software agent that communicates autonomously on social media with the task defined by the owner.

Trolling is a broad term that includes various forms of online misbehaving activity ranging from deceive and misleading comments to offensive and threatening behavior. Trolling activity is not usually categorized as spamming. Instead, the end goal of trolls is to build up confusion and inject misinformation in the target community while the spammers have financially-driven intentions. Typical examples of trolling behavior include mocking and discrediting discussion participants, inciting and escalating arguments, and impersonating expert users while spreading bad advice and false information [4].

Undoubtedly, trolling is a critical issue that threatens the role of social media as the dominant global information dissemination platform. This makes troll detection one of the most important challenges for social media administrators. A straightforward detection approach is to rely on user feedback reports, i.e., users report abusive behavior to the system and the platform moderators carefully examine the reports before decisively suspending the suspicious user account. This approach however, has been shown to suffer from various shortcomings; (1) the method is not scalable and often burdens the platform provider with excessive costs as it requires ample amount of human resources, (2) it is not sufficiently fast enough as the approach relies heavily on human intervention and often, the intended damage is already done before the detection of the troll, (3) trolls often utilize impersonation and disguise methods thus, making it arduous for moderators to detect trolling activity.

To maximize their influence, social trolls tend to expand their pool of followers on the target social platform. This can be achieved through an array of activities discussed comprehensively in [5]. Amongst these activities, impersonation is deemed as one of the most effective approaches [6]. This act can be conducted either through profile cloning [7] or fake profile identities [8]. In the former case, the trolls clone well-known profiles and try to gain followers especially among new users of the platform. In the latter, the trolls introduce a fake identity as an important person (e.g. a non-existent celebrity) or an expert (e.g. a physician) to attract followers. To reach out to a larger audience, some of these trolls, which we refer to as *fickle trolls*, go the extra mile to change their fake identity frequently [9]. In Section 3, we discuss a case study describing this type of trolling in detail.

While many research studies have focused their attention on troll detection approaches in online social platforms, they usually fall short when dealing with fickle trolls in large-scale social networks:

1. The approaches usually utilize machine learning tools to extract and analyze a set of features for detecting trolling activities. As an example, Botometer^1^ is a machine learning framework that extracts and analyses a set of over one thousand features. Clearly, these approaches are slow and impractical in very large networks with rapidly changing data.
2. The existing approaches are not specifically designed to detect fickle trolls. That is to say, fickle trolls can evade existing detection approaches due to the high frequency at which they change their identity.

This paper aims to fill this gap by introducing a method to detect fickle trolls in large-scale social networks. Considering a large dataset of user activities in an online social platform, we first extract a graph-theoretic model based on the data and then we discuss fickle troll detection in different scales. The main contributions of the paper are listed as follows:

- Firstly, we consider a single powerful machine that has the memory capacity of an entire dataset, i.e., a small size dataset. We show that the asymptotic time complexity of the centralized single-machine detection algorithm is large for early troll detection in large-scale social platforms with billions of users.
- We then discuss a streaming approach on a single machine for cases in which the dataset is larger than the memory and the data is fed to the machine sequentially. We prove that the streaming-based approach is not practical in many real-world scenarios.
- Next, we propose a massively parallel approach that is both flexible and scalable to handle an extra-large amount of data that changes over time.
- Finally, rigorous evaluations based on real-world traces (Twitter Dataset of Russian troll accounts publicly disclosed by U.S. Congress investigation [10]) are conducted to validate the efficiency of the proposed method. Our approach can detect suspicious fickle trolls approximately 6 times faster and with $50\%$ lower overhead.

To the best of our knowledge, this is the first work that focuses on fickle trolling on social networks and presents detection approaches for this unwanted and sometimes hazardous phenomenon. Every troll detection approach in a social network suffers from reactive and countermeasure methods, conducted by the trolls, either through dissembling or luring the detection mechanism. As such, these approaches and mechanisms should be revised regularly to adapt to the dynamic nature of trolling activities. In this regard, our approach is not an exception. To effectively detect fickle trolls, the proposed mechanism and the regarding parameters should be adapted frequently.

The rest of the paper is organized into five sections. The first section highlights the importance of fickle troll detection and the main contributions of this research paper. This is followed by related works in the second section. The three different methods for detection of suspicious nodes is discussed in the third section. The fourth evaluates the proposed methods and compares the results with other similar approaches. Finally, conclusive remarks are made in the fifth section.

## Background and related works

Many research studies have focused their attention on various aspects and challenges that online social networks face. The focus of these studies ranges from community detection [11–15], social recommender systems [16, 17], social media analysis [18–21] to misbehaviour and disruptive activities [22–25]. In particular, the topic of troll detection in online social networks has attracted many research studies in the course of the past few years [26–28]. Various studies have focused on troll detection approaches. Table 1 lists and compares recent approaches. Tomaiuolo et al. [29] surveyed troll detection and prevention approach comprehensively. Tsantarliotis et al. [4] presented a framework to define and predict trolling activity in social networks. Fornacciari et al. [5] focus on introducing a holistic for troll detection on Twitter^2^. Alsmadi [30] discussed features related to trolling activity using Twitter’s Russian Troll Tweets dataset. Other studies also focused their attention on various aspects of that dataset [31–33], some of them using Botometer which is a machine learning approach. However, Rauchfleisch et al. [34] discussed that these approaches suffer from relatively high false negatives and also false positives, especially for languages other than English. Tsantarliotis et al. [35] proposed a graph-theoretic model for troll detection in online social networks. They introduced a metric called *TVRank* to measure the severity of the troll activity with respect to a post.

**Table 1:** Comparison of various troll and bot detection approaches discussed in the literature

Ref.	Dataset	Technique	Limitation(s)
[4]	Reddit	Troll vulnerability metrics to predict a post is likely to become the victim of a troll attack.	Focuses on the contents of posts and the activity history of users; does not consider trolling behaviour directly.
[5]	Twitter	Takes Holistic approach, i.e., it considers various features such as sentiment analysis, time and frequency of action and etc.	The approach is slow since it considers a magnitude of features also it suffers from false positive detection
[30]	Twitter	Multi feature analysis, i.e., it considers the timing of tweets and the contents	It only focuses on the dataset, e.g., the usage of formal tone in trolls instead of slang and slurs
[31]	Twitter	Classification based on multiple behavioural and content-based features such as wording and hashtags or mentions	It suffers from high false positive and only concentrates on the behaviours extracted from one specific dataset
[32]	Twitter	Classification based on bot detection using Botometer and geolocation data	Inaccuracy of Botometer and the ability of trolls and bots to mask their real location

Other research efforts have been devoted to analyzing the behaviors and socio-cultural features of trolling activity and reactions of the target society. Mkono [36] studied the trolling activity on Tripadvisor^3^ which is a social platform specialized in travel and tourism. Hodge et al. [37] examined the geographical distribution of trolling on social media platforms. Sun et al. [26] studied the reaction of YouTube^4^ users to the trolls. They showed that well-connected users situated in densely connected communities with a prior pattern of engaging trolls are more likely to respond to trolls, especially when the trolling messages convey negative sentiments. Basak et al. [38] focused their attention on a specific type of trolling activity, i.e., public shaming. March [39] analyzed the psychological and behavioral background of trolling activities.

More recently, few research studies have focused on trolling activities, their detection, and prevention during the COVID-19 pandemic [40]. Jachim et al. [41] introduced a machine learning-based linguistic analysis approach to detect the so-called “COVID-19 infodemic” trolls. Thomas et al. [42] discussed a trolling activity during the recent pandemic. Sharma et al. [43] analyzed disinformation campaigns on major social media platforms conducted by social trolls during the COVID-19 pandemic.

In spite of the above effort, detection methods for fickle trolls have not been fully investigated in the literature. Specifically, the existing methods can not be altered to detect such activity since the main aim of the fickle trolls is to maximize their followers and thus, they may not exhibit behaviors that can be detected by typical methods. This paper aims to provide novel approaches to detect fickle trolls at different scales. We also hope that this paper provides a better understanding of this malicious behavior and serves as a basis for future investigations and research studies on this topic.

**Fig. 1:**
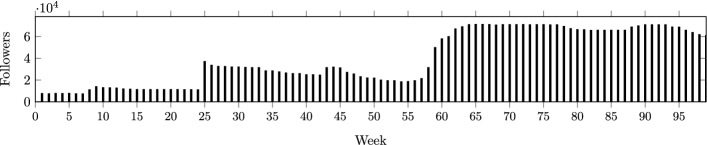
Changes in the number of followers per week for the case study

## The proposed approach

In this section, we first present a case study to clarify our approach. We then propose our assumptions and definitions before introducing the detection approaches.

### A case study

In order to gain better insights on the nature of fickle trolls, we present a case study in this section. We tracked the changes in the identity of a case study (i.e., a fickle troll) along with the topics (hashtags) and contents they posted on Twitter. We tracked this case for 97 consecutive weeks, where we gathered and logged the aforementioned data on weekly basis. At the start of the study, the troll had approximately 7k followers, and at best, they reached over 71k followers. Figure 1 shows the number of their followers per week. The troll changed their identity 4 times during the study. At week 8, the troll changed their identity to a female ship’s crew member. A major naval incident in the previous week killed many crew members onboard a ship causing public grief. During a massive wildfire (around week 25), the troll purged all the previous posts, changed their identity to a male firefighter, and posted many daily fire fighting photos. The troll changed their identity to a female environmental activist at week 43. Again they cleared all the previous history and the number of followers rose to 31k. The last change occurred at week 59 (around the same time the COVID-19 pandemic was declared by the World Health Organization). This time, they changed their identity to a female front-line health worker. The number of followers rose to 71k. The troll started posting anti-vaccination posts and hoaxes around week 80.

### System model and assumptions

We consider that the fickle trolls are resourceful, i.e., they can alter their identity without any restrictions or concerns. We consider that the system has access to user personal information. Therefore, information such as the user’s gender or job is either claimed by the user or can be deduced by content analysis. We define these as user attributes. For example, in our case study, user *x* has attributes job:firefighter, job:shipcrew, job:nurse, gender:male and gender:female. A trivial solution in this example would be to detect conflicting attributes and label them as suspected. This is possible for some of the well-known multi-valued or binary attributes. However, in ever-increasing polarized social networks [44, 45] with a wide variety of discourses, discussion topics, and trends, the realization of conflicting attributes is not feasible. Our main idea is to find users with an arbitrary and somewhat unique set of attributes in the entire network and label them as *suspected* for further investigations. To this end, we construct a graph where nodes are users or attributes. The edges show relations between users and attributes. In what follows, we define the attribute graph formally and then discuss its characteristics.

**Fig. 2:**
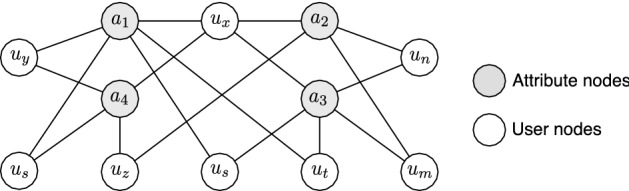
An example of an attribute graph

### Attribute graphs

Consider *G*(*V*, *E*) to be a simple undirected graph, where the set *V* represents vertices that are comprised of two types: users with label $u_i$ and attributes denoted as $a_j$. Each subset is denoted by $V_u$ and $V_a$, respectively, and $|V| = n$. In this graph, an edge exists between $u_i$ and $a_j$ if the user $u_i$ has the attribute $a_j$. We call this the attribute graph. Suppose that $\Delta _{a}$ and $\Delta _{u}$ represent the maximum degree of attribute nodes^5^ and user nodes, respectively, and $\Delta _{u}\gg \Delta _{a}$. Also, let *d* be the diameter of the graph and $A_{u_i}$ be the neighboring set of the node $u_i$. Figure 2 exemplifies such a graph, where labels $\{u_1, u_2, u_3, u_4\}$ stand for users and $\{a_1, a_2, a_3, a_4\}$ signify the attributes.

#### Clustering coefficient

Clustering coefficient is a measure of the degree to which nodes in a graph tend to cluster together. It is usually represented by a real value between zero and one, i.e., zero when there is no clustering at all and one for total clustering. In graphs where the value is close to one, the nodes tend to create tightly knit groups characterised by a relatively high density of ties, whereas when it is close to zero, the nodes form looser clusters among each other. This notion is often interpreted as the probability that two incident edges are completed by a third one to form a triangle. Clustering coefficient is one of the important measures of the performance of massively parallel approaches [46, 47]. In what follows, we show that this measure is zero for the attribute graph. The proof is based on the following lemma:

##### Lemma 1

In an attribute graph *G*(*V*, *E*), every cycle has an even number of nodes.

##### Proof

Suppose that a cycle in an attribute graph has odd number of nodes. Then, either two nodes in $V_u$ or two nodes in $V_a$ become neighbors in the cycle, which contradicts the definition of attribute graphs.$\square$

With no triangles to form clusters, the clustering coefficient of the attribute graph is equal to zero. As we will show later in this paper, this is actually an important measure for our proposed detection methods.

#### Suspected nodes

When there are only one or few users with exactly the same set of attributes i.e., there are one or few user nodes that are connected to the same attributes in the graph, we consider these nodes as suspected nodes. Note that in here, by few users, we mean the number of users that is smaller than a system-wide threshold, denoted by $\tau$ and also we ignore user nodes with attributes smaller than $\delta$ which is also a system-wide threshold. The actual value of these thresholds depends on the social network.

**Fig. 3:**
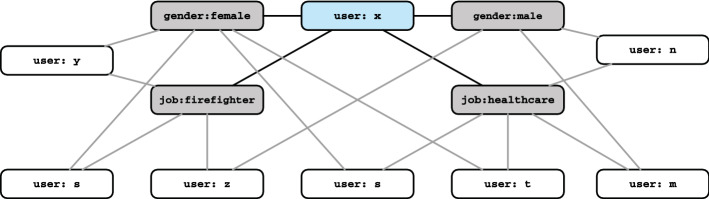
Another example of an attribute graph

Figure 3 shows an example of such a scenario. As shown in the figure, a user user:x is connected to four attributes and no other user has exactly this set of attributes. This makes the user unique in this respect. Thus, we consider it as a *suspected* node.

### Detection methods

In this paper, we discuss possible detection methods at three different scales:

1. *Single Machine*: In this approach, we have a single computation machine and the whole attribute graph is available inside the machine. We consider the memory of the machine to be large enough to store the attribute graph. Although this assumption is unrealistic for most real-world networks, there are however, some cases where the graph does not exceed the size of the machine’s memory. We also suppose that the attribute graph remains fixed during the computation. The machine processes the graph and detects the suspected nodes.
2. *Streaming*: In this approach, we have a single computation machine with limited memory, denoted by *m*. The input of the machine is a sequence of edges that are streamed to the machine. Linear scanning of the memory is possible for the processor. Here, the goal is to minimize *m*.
3. *Massively Parallel*: In this approach, we have *M* machines that work in parallel and are interconnected. Nodes of the graph are partitioned inside the machines. Edges whose end nodes reside in different machines are called *outbound* edges as opposed to *inbound* edges. The machines communicate with each other using a message passing approach. The goal here is to minimize the number of transmitted messages between machines.

### Single machine approach

By our definition, if there are fewer than $\tau$ nodes that have exactly the same neighbouring set, those nodes are considered as suspected. Here, we have a single processing machine with a memory large enough to load the entire attribute graph. We are interested in an algorithmic approach to explore suspected nodes. In what follows, we present such an approach. Lemma 2 establishes the basis for a modified version of the well-known Depth-First-Search (DFS) algorithm to detect such nodes.

#### Lemma 2

Consider two nodes $u_x, u_y \in V_u$. If every 4-cycle^6^ that starts from $u_x$ either passes through $u_y$ or passes through attributes connected to $u_x$, then $A_{u_x} \subseteq A_{u_y}$.

#### *Proof*

Suppose that $u_x$ has another attribute in its neighboring set $a_i$ such that $a_i$ is not in the neighboring set of $u_y$. Then, the 4-cycle that starts from $u_x$ and passes through $a_i$ cannot pass through $u_y$ since they are not connected. Thus, in this case there is at least one 4-cycle that starts from $u_x$ and does not pass through $u_y$, which is a contradiction.$\square$

If $A_{u_x} = A_{u_y}$, i.e., the two nodes have the same attribute sets, and if there are greater than $\tau$ such nodes, then neither is suspected.

The algorithm to find all 4-cycles starting from a node can be obtained with minor alterations to the well-known DFS algorithm. The number of iterations for the algorithm in this mode is $\mathcal {O}(n^3)$ which is impractical even for medium-sized graphs. On the other hand, the storage requirement for this approach is $\mathcal {O}(|V|+|E|)$. Again, when there are millions of nodes with billions of edges between them (as evident in most online social networks), fitting the entire graph inside the memory of a single machine is infeasible.

To speed up the algorithm, one approach is to utilize multi-threading, where each thread chooses a start point and executes the DFS algorithm. The threads stop when all nodes are visited by at least one thread and at least once. This approach can be performed either synchronously or asynchronously. Synchronous mode requires heavy coordination between threads which makes it impractical. Asynchronous mode is simpler to implement and does not have the coordination overhead, however, it may over-count cycles, i.e., a thread counts a cycle that was already counted by another thread.

### Streaming

To be able to handle the large size of the data that often goes beyond the memory capacity of a single machine, another strategy is to stream the data gradually to the machine. In the streaming approach, the machine processes the input in a multi-pass manner. The number of times that the machine linearly scans the memory is an important measure for the performance of any streaming-based processing approach [48]. The size of available memory to the machine is limited. So, as the new inputs from the stream are received by the machine, the oldest ones are overwritten. Thus, the minimum required memory is also another important performance measure.

**Fig. 4:**
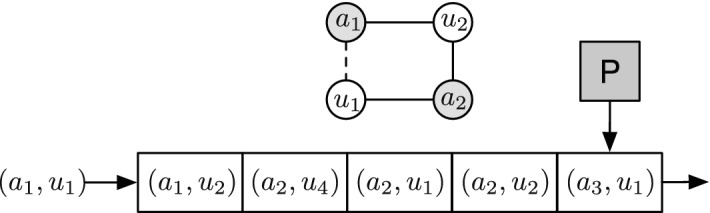
The concept of streaming approach

The input data in this approach is a sequence of edges. Although the node-based streaming is also possible, in this paper, we focus on edge-based streaming which is illustrated in Fig. 4. The figure illustrates a streaming scenario, i.e., edges are fed (from left side) to a buffer with limited capacity (in this example the capacity is 5). The processor (P) in the figure scans the memory and constructs cycles. The processor is able to scan the memory indefinitely, however, as the buffer is capped, edges are deleted from the buffer (from right side). In the first pass, P forms the incomplete cycle of $a_1, u_2, a_2, u_1$. In the second pass, $(a_1, u_1)$ is added to the memory thus, resulting in the formation of a 4-cycle. There are many possible patterns for the sequence of edges, two of which are described below.

#### Random sequences

In this pattern, the edges are received by the machine uniformly at random. This approach is useful for cases wherein the graph is highly flexible and changes during execution. Nonetheless, it uses $\mathcal {O}(n)$ memory space in the worst-case which makes it less attractive for large volumes of data.

#### Deterministic sequences

There are various deterministic sequencing patterns. Here, we adopt the Breadth-First-Search (BFS) algorithm to determine the sequence in which edges are sent to the machine. The first node is selected randomly, then the edges connected to that node are sent, and so on and so forth. Theorem 1 determines the memory space used inside the machine in the worst-case along with the number of passes the machine scans the memory.

##### Theorem 1

The BFS-based deterministic streaming approach uses $\mathcal {O}(\Delta _{u})$ memory space with $\mathcal {O}(d\Delta _{u})$ passes.

##### *Proof*

The proof is straightforward. In order to detect 4-cycles, the algorithm needs to store 4 levels of the BFS algorithm. The first level is a node from $V_{a}$, the second level has at most $\Delta _{a}$ nodes, the third has at most $\Delta _{u}\Delta _{a}$ and the forth level has $\Delta _{u}\Delta ^{2}_{a}$ nodes, thus $\mathcal {O}(\Delta _{u})$ for memory space. After formation of the 4 levels, a DFS can find the suspected nodes. There are $\mathcal {O}(\Delta _{u})$ nodes and at most $\mathcal {O}(\Delta _{u})$ edges and at most *d* levels, thus requiring $\mathcal {O}(d\Delta _{u})$ passes.$\square$

This approach is practical when the maximum degree $\Delta _{u}$ is at most logarithmic; however, this is not the case for many real-world applications. For example, at least half of the users are either male or female, i.e, $\mathcal {O}(n)$. Moreover, the processing takes considerable amount of time which is not feasible for most real-world cases. To overcome these issues, we employ the following massively parallel approach.

### Massively parallel approach

We utilize a vertex-centric distributed approach [49] for the detection of suspected nodes when there are large number of nodes in the attribute graph. The basic idea in the vertex-centric distributed approach is to iteratively execute an algorithm over vertices of a graph. In this setting, the vertex-centric algorithm in each node includes data from adjacent vertices or incoming edges as input, and the produced output is communicated along outgoing edges of the vertex. These algorithms are usually iteratively executed for a predefined number of times or until they converge to the desired properties. The distributed algorithm is performed inside an array of trusted machines that are interconnected together and communicate with each other using message passing or via memory sharing. The vertices of the graph are distributed inside those machines prior to the execution (which is called the placement phase) and the inbound and outbound edges are established, then the vertex-centric algorithm is executed on each vertex in each machine.

Various large-scale parallel approaches have been proposed for graph processing. Since the introduction of Pregel [50] by Google, many other techniques such as GraphLab [51], GraphX [52] and Pangolin [53] have been reported in the literature. Some of these approaches are vertex-centric while others are edge-centric. Hybrid approaches have also been proposed [54]. Other studies have focused on improving the performance of these approaches [55]. It has been observed that usually the input graphs to massively parallel graph processing tools are preferential attachment networks which are categorized as scale-free graphs [56, 57]. These types of graphs exhibit two major properties as given below.

#### Power-law degree distribution

Power-law degree distribution means that a small fraction of nodes in the graph have many direct neighbors while the rest of the nodes have few neighbors. For example, one percent of the nodes in the Twitter’s web graph are adjacent to nearly half of the edges. This causes a series of issues in any massively parallel approach when applied on such graphs. It leads to imbalance in the computation, network traffic, and storage of machines that contain nodes with higher degrees. To remedy this issue, PowerGraph [56] and its successors such as PowerLyra [58] differentiate the functionality of nodes, i.e., the high degree nodes (nodes with many neighbours) perform series of actions that are different from low degree nodes (nodes with fewer neighbours).

As the interaction between users and attributes follows the preferential attachment scheme [59], it is sound to assume that the attribute graph also follows the power-law degree distribution. PowerGraph performs well in this scenario; however, the attribute graph has another property (low clustering coefficient) that distinguishes it from typical scale-free graphs and leads to poor performance of PowerGraph and PowerGraph-like approaches on the attribute graph.

#### High clustering coefficient

As discussed earlier in this paper, in a vertex-centric approach with multiple machines, the first task is to distribute nodes among those machines. A number of approaches have been proposed for node placement that ranges from fully random placement to greedy algorithms. After the placement in each machine, a local graph is constructed among the nodes that reside in the machine. In PowerGraph, instead of nodes that are placed in other machines, exact “replicas” of those nodes are placed in each machine. This approach is indeed feasible for scale-free graphs. High clustering coefficient means that the probability that two connected nodes are also connected to the same replica is high. This means that the number of replicas reduces with high probability.

**Fig. 5:**
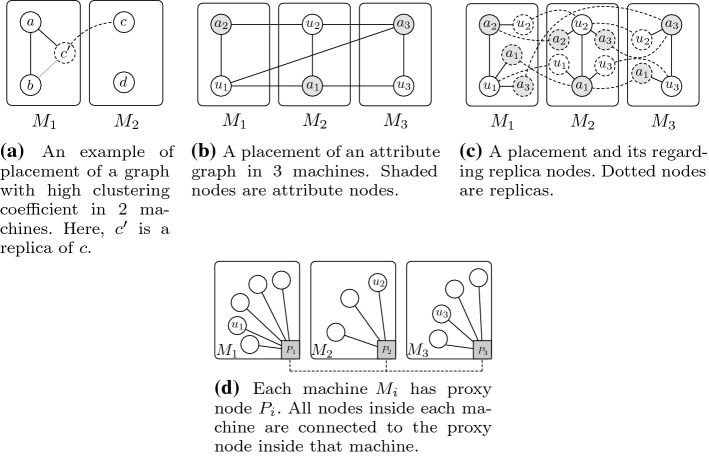
Various examples of node placement inside machines

To clarify the above argument, consider the example depicted in Fig. 5a. Suppose that we have a graph with high clustering coefficient and we distribute the constituent nodes of this graph between two machines, namely $M_1$ and $M_2$. Let us also assume that two nodes *a* and *b* of the graph are connected and reside in the first machine and *nodec* is connected to *a* and resides in another machine. Using the above approach, we make a replica of *c*, say $c'$, and assign it to the first machine ($M_1$) and then construct the edges, i.e., an inbound edge between *a* and $c'$ and an outbound edge between *c* and $c'$. High clustering coefficient means that if *a* and *b* are connected and *a* and *c* are connected, then it is highly probable that *b* and *c* are also connected, which implies that the inbound edge between *b* and $c'$ is formed with high probability. Unfortunately, this is not valid for the attribute graph as its clustering coefficient is equal to zero. For example, if a user node and an attribute node are placed inside a machine and the two nodes are connected, and the user node is also connected to another attribute node, then according to definition, it is not possible for the two attribute nodes to have an edge between them. This renders replica-based approaches extremely inefficient for attribute graphs. To further exemplify, suppose that we want to distribute an attribute graph over 3 different machines, namely $M_1$, $M_2$, and $M_3$. One possible placement for this scenario is depicted in Fig. 5b. Figure 5c shows the replicas that are created in each machine to construct the graph. Each node inside the machine is connected to a replica of its original neighbour and replicas are connected to their respective original nodes. Clearly, the amount of outbound connections and the number of replicas in each machine make this approach inefficient.

The above argument justify that the existing approaches are not suitable for attribute graphs. As such, we introduce an approach that is specifically tailored for attribute graphs. The approach consists of the following three steps for node placement:

1. User nodes are distributed based on a balanced hash function.
2. Each machine contains a node called the proxy node of the machine, denoted by $P_i$, where *i* is the index of the machine.
3. Star-like connection is established between user nodes inside each machine and the proxy node.

Figure 5d shows an example of our proposed node placement scheme in which we assume that:

- Each user node has a list of its neighbors, i.e., its attributes.
- Nodes are placed inside machines based on the hashes of their IDs.
- Outbound communication is only possible through proxy nodes.
- The execution can be done either synchronously or asynchronously.
- The user nodes are dynamic, i.e., new nodes can be added to the system or old ones may be deleted from the system at any time.
- Machines are dynamic, i.e., new machines are added to the system to improve scalability. Old machines may be merged to reduce overhead.

Building on these assumptions, we present our vertex-centric algorithm to find suspected nodes in what follows.

### Detection algorithm

Essentially, what we want here is for a node such as $u_i$ to have the neighboring set of every node that has common attribute with $u_i$. For example, in the attribute graph of Fig. 2, we want $u_s$ to have the neighboring set of $u_x$ and $u_y$ since they have common attributes, i.e., $a_1$ and $a_4$ with $u_s$. The straightforward approach would be for each node to determine its neighboring set and send it to all its two-hop neighbors. In a distributed vertex-centric with multiple machines, each vertex is not aware of the neighboring set of its two-hop neighbors after the placement. To do so, every user node $u_x$ constructs a beacon $<u_x, a_i>$ for each of its attributes and sends each of them to the proxy node of the machine. Upon receiving the beacon, the receiver broadcasts the beacon to all other proxy nodes. Each proxy node relays the beacons locally to all of its neighbors. The receiver (e.g. $u_y$) looks up its neighboring list and if it has $a_i$, sends back an acknowledgment response to the proxy node. The proxy node routes back the response to the sender’s proxy node. The sender’s proxy node then relays the acknowledgment (ACK) to the sender. The sender saves $<u_y, a_i>$ locally. In this way, each node finds the neighbors of its attributes. For example, for the graph given in Fig. 2, node $u_2$ receives tuples $<a_1, u_1>,<a_2, u_1>, <a_3, u_1>$ and so on. The pseudocode of this entire procedure is given in Algorithm 1.

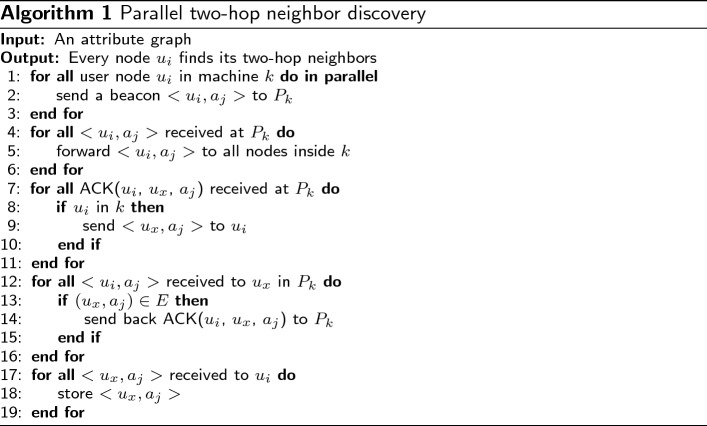



The execution of the algorithm finishes when every beacon is sent and received by its destination. After the execution, each node has the neighboring sets of its two-hop neighbors. Using the list, each node can determine whether it is suspected or not. For example, consider that the algorithm is executed on the attribute graph presented in Fig. 2 and $\tau$ is 2. After the execution, the system finds out there are less than $\tau$ nodes that has $a_1$, $a_2$, $a_3$ and $a_4$ as attributes and thus, $u_x$ is marked to be suspected.

### Complexity analysis

The most important measure in these type of approaches is the amount of outbound communication in terms of the number of transmitted messages between machines which is also called transmission overhead. This is mainly due to the fact that usually this type of communication incurs higher delays which in turn reduces the system’s overall performance. The transmission overhead for Algorithm 1 is $\mathcal {O}(|V_u||V_a|)$ in asynchronous mode. To improve the algorithm and reduce the overhead, a batching approach can be utilized in proxy nodes. In this manner, the algorithm works synchronously at each step, i.e., the proxy node waits until it receives all beacons, batches them, and then sends the batch to other proxy nodes. Although the size of the transmitted message is still the same, the number of transmission attempts reduces significantly and thus, the delays imposed by the network reduces drastically. However, if one or more beacons are not received by the proxy due to an unforeseen circumstance such as system fault, all other messages have to wait until a predefined timeout, which may result in higher delays.

## Evaluation

In order to evaluate the performance of the proposed methods, all three approaches introduced in this paper were implemented. For the single machine and the streaming approaches, experiments are performed on a dedicated server that has 4 Intel Xenon cores and 64GB DRAM. For the massively parallel approach, we utilized a cloud-based approach with up to 32 VM instances each with 16GB of DRAM and 2 Intel Xenon cores. The operating system of each VM was Linux-based and the instances were connected to each other over 1Gb Ethernet links. We implemented the approaches using the Ruby programming language.

Numerous validation experiments have been established. However, for the sake of specific illustration, validation results are presented for limited number of scenarios. We adopted 95 percent confidence level to make sure that, on average, the confidence interval which is calculated using *t*-student distribution and standard error contains the true values around 95 percent of the time.

For the sake of evaluation, we used a Twitter dataset of Russian troll accounts publicly disclosed by U.S. Congress investigation [10]. The dataset contains over 43 million elections-related posts shared on Twitter between September 16 and October 21, 2016, by about 5.7 million distinct users and their various social interactions such as re-tweeting, replying, mentioning, etc.

**Fig. 6:**
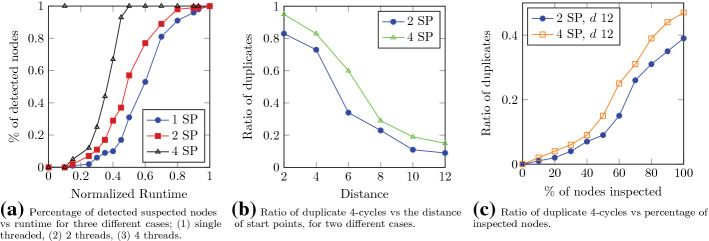
Evaluation of single machine approach

### Single machine approach

We evaluated the performance of the single machine approach using the aforementioned dataset.

#### Convergence rate

Figure 6a compares the rate of convergence for three different cases. The *Y* axis shows the percentage of the detected suspected nodes and *X* represents the normalized runtime. The first case is single threaded. In this case, a node was selected at random, after which we executed the algorithm using a single thread. In the two latter cases, two and four threads were executed, respectively, where the start points were also selected uniformly at random. We observe that with increase in the number of independent threads, the rate at which the 4-cycles are being detected also increases. Thus, as the detection rate rises, it leads to growth in the rate of convergence of the algorithm.

#### Overcounting

In the case of multiple threads, one common scenario is overcounting the 4-cycles. One approach to prevent this is synchronization between multiple threads which is costly and time consuming. A common approach in these cases is to execute threads independently and discard the duplicates. In what follows, we discuss the performance of the single machine approach from the perspective of overcounting.

In Fig. 6b, the effect of start point distances on the overcounting is investigated. The figure plots the ratio of duplicate 4-cycles detected by threads when the distance between start points increases for two cases; (1) two threads and (2) four threads. The number of duplicates decreases as the distance between start points increases. In the case of four threads, the ratio of duplicates is slightly higher since the probability of overcounting increases. Obviously, the distance is bounded by the diameter of the attribute graph.

We are also interested in the rate at which the ratio of duplicates grows when the algorithm progresses as depicted in Fig. 6c. The *Y* axis represents the ratio of duplicate 4-cycles and the *X* axis shows the percentage of inspected nodes. In both cases, we assumed the distance to be equal to 12. In the early stages of the algorithm, the rate at which the overcounting occurs is significantly lower than the later stages and this rate is higher for the case of 4 threads.

**Fig. 7:**
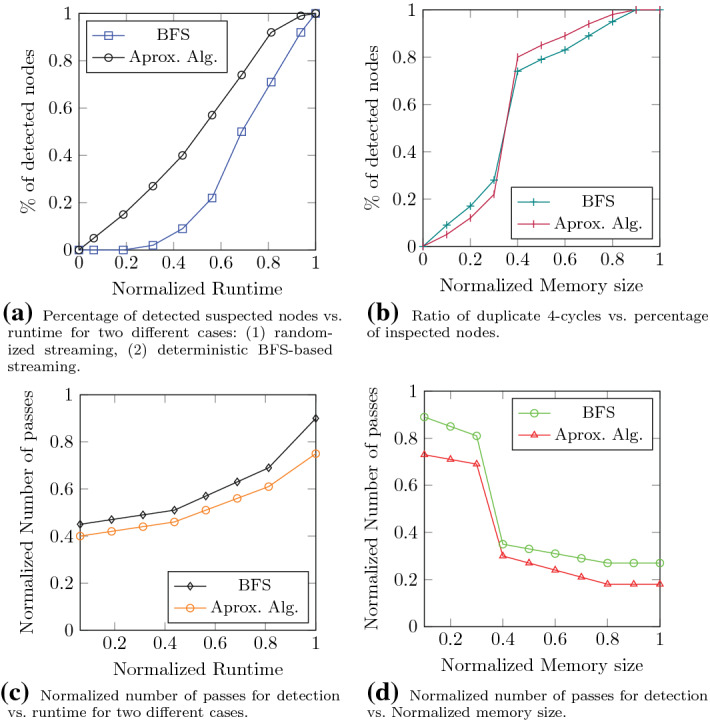
Evaluation of the streaming approach

### Streaming approach

Figure 7 depicts the results obtained for the streaming approach.

#### Convergence rate

We examined the convergence rate of the streaming approach for the given dataset for two different cases, i.e., random streams and deterministic BFS streams. Here, we consider the memory large enough for the algorithm to be able to detect all the suspected nodes. The results are plotted in Fig. 7a. As shown in the figure, the deterministic approach outperforms the random approach.

Figure 7b investigates the effect of memory size on the convergence of the streaming approach. Here, we consider the maximum memory size to be equal to the size of the entire graph and thus, plotted the normalized memory size accordingly. When the memory size is small, the chance that the algorithm misses a 4-cycle increases since one or more edges of the cycle may be truncated from the memory before being included in the algorithm to form a cycle. As the memory size grows, the number of detected nodes rises. We also observe a threshold which in this case is around 40% of maximum memory size. This threshold is related to $\Delta _{u}$ as discussed earlier in this paper, i.e., when the memory size is smaller than the threshold, some of the edges are being evicted before the formation of 4-cycles which consequently, disrupts the detection.

#### Number of passes

We have already seen that in a streaming approach, the algorithm scans the memory for a number of times to detect 4-cycles. Figure 7c depicts the normalized number of passes when the algorithm progresses. The rate of growth increases as the algorithm advances mainly due to the fact that the number of constructed incomplete cycles grows and thus, the algorithm needs to re-scan the memory more often.

The effect of memory size is also investigated in Fig. 7d. As the memory size grows, the number of edges that the algorithm scans in each pass grows and thus, the total number of passes reduces. Again, a threshold phenomena can be seen in the figure. In both figures, the number of passes for the deterministic BFS case is lower than the random sequence as the incoming stream for the latter is uniformly random and thus, for the construction of a cycle the algorithm may need more passes to find missing edges.

### Massively parallel approach

**Fig. 8:**
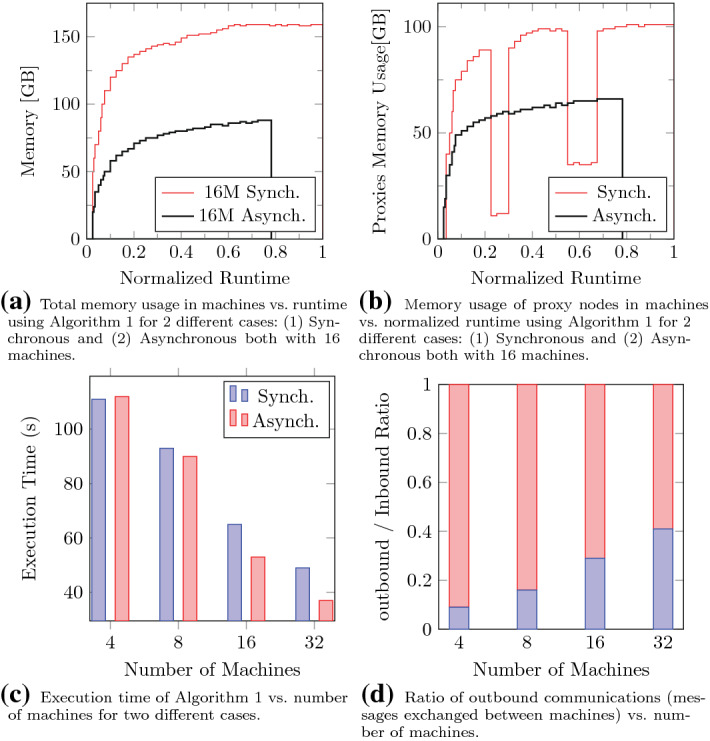
Evaluation of the proposed massively parallel approach

Figure 8 shows the evaluation of our proposed massively parallel approach.

#### Memory usage

Figure 8a depicts the total memory usage of the proposed method executed in parallel over 16 exactly same machines. Note that in this figure, we aggregated the memory usage of all 16 machines. We considered two different cases: (1) synchronous proxies and (2) asynchronous execution. As shown in the figure, the memory usage grows as the algorithm progresses. The asynchronous approach has significantly lower memory usage compared to that of the synchronous approach mainly due to the delay imposed for synchronization inside proxy nodes.

We also examined the memory usage of only the proxy nodes in Fig. 8b. Here, the evaluation settings were the same. Comparing the figures, we realize that the proxy nodes impose most of the memory burden on the machines. The two gaps in the synchronous execution is related to the phases in which proxies gather inbound messages and send them to other proxy nodes.

#### Parallelism

Figure 8c shows the impact of parallelism on the performance of the proposed approach both in synchronous and asynchronous executions. As the number of machines increases, the execution time of both cases reduces; however, the parallelism has greater impact on the asynchronous case. Generally, the asynchronous execution experiences lower delays and thus, outperforms the synchronous approach.

One important factor to decide whether to use the synchronous or the asynchronous approach is the ratio of outbound to inbound communications. All the previous arguments have shown that the asynchronous approach outperforms the synchronous approach, i.e., both showing lower memory usage and lower execution time. However, from the communication perspective, the synchronous approach has significant superiority to the other approach. Figure 8d shows the ratio of outbound to inbound communications (lowers are outbound) for the case of asynchronous approach. As the outbound communications for the synchronous approach is negligible, it has not been shown in the figure.

The synchronous execution is best fitted for cases in which the bandwidth between machines are low or the communication link is unstable, lossy or with unpredictable delays. Obviously, these are not the cases for cloud infrastructures. But there may be some use cases for which the detection algorithm must be executed over the edge-computing environments.

#### Comparison with PowerGraph

In order to compare our approach, we implemented a two-hop neighbor discovery algorithm using PowerGraph. Table 2 compares the execution time and maximum memory usage of our synchronous parallel approach with PowerGraph for various number of machines. The table lists 3 different scenarios, with few number of processing machines ($M=4$), $M=8$, and massively parallel one ($M=16$). In each scenario, the execution time reduces significantly when the degree of parallelism increases. Nonetheless, with the same attribute graph, the execution time of PowerGraph is significantly higher mainly due to the overhead caused by replicas. These replicas not only escalate the outbound traffic which increases the execution time of the algorithm, but also cause more memory overhead in the PowerGraph implementation.

**Table 2:** Comparison of our approach with PowerGraph. Here, *M* represents number of machines

		$M=4$	$M=8$	$M=16$
PowerGraph	Exec. Time (s)	749	534	313
	Max. Memory (GB)	743	612	509
Proposed approach	Exec. Time (s)	107	88	59
	Max. Mem. (GB)	340	229	159

## Conclusion

The large-scale and highly dynamic nature of social networks necessitates specifically tailored troll detection methods. In this paper, we investigated a massively parallel approach to detect fickle trolls in large-scale social networks. We first proved that centralized and streaming approaches are not practical in real-world large-scale networks as they are slow for early detection of these trolls. We then proposed a parallel detection approach that uses a vertex-centric parallel two-hop neighbor discovery algorithm. Our evaluations based on real-world traces confirmed that our proposed method can outperform similar parallel approaches by order of magnitude. Our future direction aims at utilizing fast machine-learning approaches to prune the attribute graph (and thus, minimize the overheads incurred) to further enhance the proposed detection algorithm. We will also consider using an optimization algorithm to solve the problem of excessive computation time and load balancing in our proposed parallel approach in the future.

## Data Availability

The dataset used and/or analyzed in the current study is available at https://www.recode.net/2017/11/2/16598312/russia-twitter-trump-twitter-deactivated-handle-list. Results sets are available from the corresponding author on reasonable request.

## Footnotes

1 https://botometer.osome.iu.edu/.

2 https://twitter.com.

3 https://tripadvisor.com.

4 https://youtube.com.

5 In this paper, the terms node and vertex represent exactly the same notion and have been used interchangeably.

6 A cycle with *x* nodes is called an *x*-cycle.
